# Effects of Understory Vegetation and Litter on Plant Nitrogen (N), Phosphorus (P), N∶P Ratio and Their Relationships with Growth Rate of Indigenous Seedlings in Subtropical Plantations

**DOI:** 10.1371/journal.pone.0084130

**Published:** 2013-12-27

**Authors:** Jun Wang, Dafeng Hui, Hai Ren, Zhanfeng Liu, Long Yang

**Affiliations:** 1 Key Laboratory of Vegetation Restoration and Management of Degraded Ecosystems, South China Botanical Garden, Chinese Academy of Sciences, Guangzhou, China; 2 Department of Biological Sciences, Tennessee State University, Nashville, Tennessee, United States of America; 3 Centre of Resource and Environment, Guangzhou Institute of Geography, Guangzhou, China; Wuhan Botanical Garden, Chinese Academy of Sciences, China

## Abstract

Establishing seedlings in subtropical plantations is very important for forest health, succession and management. Information on seedling nutrient concentrations is essential for both the selection of suitable indigenous tree species to accelerate succession of the established plantation and sustainable forest management. In this study, we investigated the concentrations of nitrogen ([N]), phosphorus ([P]), and N∶P ratio in leaves, stems and roots of seedlings of three indigenous tree species (*Castanopsis chinensis*, *Michelia chapensis* and *Psychotria rubra*) transplanted with removing or retaining understory vegetation and litter at two typical subtropical forest plantations (*Eucalyptus* plantation and native species plantation). We also measured the relative growth rate (RGR) of seedling height, and developed the relationships between RGR and leaf [N], [P] and N∶P ratio. Results showed that treatments of understory vegetation and associated litter (i.e. removal or retained) generally had no significant effects on leaf [N], [P], N∶P ratio and RGR of the transplanted tree seedlings for the experimental period. But among different species, there were significant differences in nutrient concentrations. *M. chapensis* and *P. rubra* had higher [N] and [P] compared to *C. chinensis*. [N] and [P] also varied among different plant tissues with much higher values in leaves than in roots for all indigenous species. RGR of indigenous tree seedlings was mostly positively correlated with leaf [N] and [P], but negatively correlated with leaf N∶P ratio. Considering the low [P] and high N∶P ratio observed in the introduced indigenous tree seedlings, we propose that the current experimental plantations might be P limited for plant growth.

## Introduction

Currently in South China, large areas of plantations established in the past several decades still have a simple structure with few typical zonal forest species in the understory[1], [2]. For this reason, there is always a need to promote environmental conditions for the established plantations to support species assemblages similar to natural forests. Because colonization of indigenous species into such plantations is limited by a lack of seed source [3], [4], introducing seedlings of the desired species seems to be the most reliable approach in silvicultural practices [5].

Nutrient concentrations, especially of nitrogen (N) and phosphorus (P), play a great role in the machinery of photosynthesis, cell growth and metabolism, and can limit plant growth in most terrestrial ecosystems [6], [7]. For example, leaf nitrogen concentration [N] is an important determinant of photosynthetic capacity [8], while phosphorus concentration [P] regulates energy storage and tree carbon gain. As indicated by previous studies, nutrient concentrations of plants mostly vary with their nutrient uptake capability among specie [9], [10]. From this viewpoint, to improve early seedling establishment and accelerate colonization of indigenous species into plantations, transplanting seedlings of species with a greater nutrient uptake potential or capacity is crucial.

In addition to the genetically fixed nutrient uptake potential, the ability of a plant to capture nutrients is often controlled by many biotic and abiotic factors [11], [12]. Good site preparation can enhance plant nutrient uptake and improve seedling performances [13]. As for the introduced seedlings in the understory, removal of existing understory vegetation may be beneficial for seedling growth due to reducing competition for resources such as light and soil nutrients. For example, Parker et al. (2012) demonstrated that controlling herbaceous vegetation can increase leaf [N] and [P] of eastern white pine [14]. However, such treatment effects may differ depending on the variation of understory vegetation type and the intrinsic trait of introduced species.

Soil fertility directly influences plant nutrient concentrations, and reduced mineral nutrient supply often leads to limited capacity of forest regeneration [7], [15]. Güsewell (2004) found that plant N∶P ratios can influence the functioning of terrestrial vegetation such as the growth of individual plants, and serves as a useful indicator for the shift between N- and P-limitation [8]. The RGR of transplanted indigenous tree seedlings under the subtropical plantations could be related to leaf [N], [P] and N∶P ratio. For example, Peng et al (2011) have demonstrated that under nutrient-enriched conditions, RGR for the shrubs positively correlated with N concentration and N∶P ratios, but was not significantly related to P concentration [16]. Therefore, understanding the variation of N∶P ratio of the transplanted indigenous seedlings is important for decision makers and silviculture practices for the subtropical plantations.

In this study, we investigated [N], [P], and N∶P ratio in the leaves, stems and roots of seedlings of three indigenous tree species (i.e. shade-intolerant *Castanopsis chinensis*, intermediate shade-tolerant *Michelia chapensis* and shade-tolerant *Psychotria rubra*) in South China under two typical subtropical plantations (*Eucalyptus* plantation and native species plantation). The main objectives of our study were 1) to assess the effects of understory vegetation and litter on plant nutrients; 2) to develop the relationships between seedling relative growth rate (RGR) and leaf [N], [P] and N∶P ratio; and 3) to provide information on selecting suitable indigenous species to enhance plant establishment under the plantations. Specifically, we sought to answer the following two questions: (1) Does removing understory vegetation and litter enhance [N] and [P] of the transplanted seedlings? (2) What are the differences in seedling nutrient uptake ability among the transplanted indigenous species?

## Materials and Methods

### Ethics Statement

The study site is maintained by the South China Botanical Garden, Chinese Academy of Sciences. All necessary permits were obtained for the described field study. The field study did not involve protected species.

### Study area

The study area is located at the Heshan National Field Research Station of Forest Ecosystem, Chinese Academy of Science (112°54′ E, 22°41′ N), Heshan City, Guangdong, South China. The area is characterized by a typical subtropical monsoon climate with a mean annual temperature of 21.7°C. The mean annual rainfall is about 1700 mm, which is concentrated between May and September. The mean annual evaporation is approximately 1600 mm, and the elevation ranges from 0 to 90 m. The soil is laterite. The zonal climax vegetation is subtropical monsoon evergreen broad-leaved forest, and the closest remnant zonal forest is located at Dinghushan Mountain, about 70 km north of the research station.

To restore the forests in the highly degraded ecosystems, experimental plantations were established in 1984 on the selected relatively homogenous hilly land. For this study, we selected two plantations: a 1.79-ha *Eucalyptus* plantation (EP) and a 2.68-ha native species plantation (NP). The main established species at EP was *Eucalyptus exserta*, with mean basal area of 24.6 m^2^ ha^−1^ and height of 12.7 m. The main established species at NP were *Schima superba* and *Cinnamomum burmanii*, with mean basal area of 32.2 m^2^ ha^−1^ and height of 11.7 m. All the trees in the plantations were planted with 2.5 m×2.5 m spacing. The two plantations had been established in areas with similar soil physical and chemical properties and aboveground vegetation [17], and then left to develop naturally without anthropogenic disturbance. Currently in Guangdong and China, it is an urgent task to accelerate the succession of established plantations (e.g. *Eucalyptus* plantation and other native species plantations) to more natural stages for providing adequate ecosystem services.

### Plant materials

The three species selected for this study are native and common in the zonal climax monsoon broad-leaved forest in South China but either absent or rare in the experimental plantations. *C. chinensis* is a relatively shade-intolerant species that can regenerate in a wide range of forest types and various light conditions from understory to large gaps [18], [19]. *M. chapensis* is an intermediate shade-tolerant tree species often found in distributed areas. As a canopy tree species, it can reach 30 m in height [20]. *P. rubra* is a shade-tolerant small tree species occurring in shady and humid microhabitats. It is often found in late successional plantations and secondary forests [18].

### Experimental design

To examine [N], [P] and N∶P ratio in the leaves, stems and roots of target species, we transplanted seedlings to the two plantations. The experiment was established using the split-plot design with the understory vegetation and litter removal as the main treatment factor, and targeted species as split plot treatment factor. The main treatments include understory vegetation and litter removed (VR), and not removed as a control (CK). We did not separate the understory vegetation and litter so both were either removed or remained in the plantations. At each plantation, three blocks were established as replications. Each block (3×4 m^2^) was then subdivided into two sections. In one section, the aboveground understory vegetation and litter were removed (VR treatment). In another section, aboveground vegetation and litter were not removed (CK as control). Within each section, three plots (1×2 m^2^ each) were established and three transplanted species were randomly assigned to one of the three plots. In the plots with the VR treatment, understory vegetation and litter were cleared by hand before seedlings were transplanted and every 2 weeks thereafter during the experiment. Each plot was surrounded by a nylon mesh fence (1-m in height) to prevent herbivory by wild animals such as rodents.

Seedlings of *C. chinensis* and *P. rubra* for transplanting in the field were grown from the seeds collected from the monsoon broadleaved forest in the Dinghushan Biosphere Reserve. Seedlings of *M. chapensis* were obtained from the Forestry Institute of Guangdong Province, China. All seedlings for transplanting were about 6 months old, and no significant differences for the initial size of the seedlings were found among the three species. Thirty seedlings of one species were transplanted in each plot after a rainfall in late April 2007. Each species was represented by two plots (VR and CK) in each block. Transplanted seedlings were watered shortly after planting but were not watered or fertilized after that during the experimental period. The experiment ended in December, 2008.

### Determination of environmental conditions

Soil properties were measured before the seedlings were transplanted. In each plantation, 10 soil cores (4 cm diameter, 0 to 20 cm depth) were collected from random sampling points and then combined to provide one composite soil sample for each block. Soil samples were air-dried and sieved for chemical analysis. Soil chemical properties including soil organic matter, hydrolyzed nitrogen, and available phosphorus were analyzed using standard methods [21]–[23]. The light intensity was measured for each block (approximately 10 measurements 1-m above the soil surface) at the two plantations between 12:00–14:00 pm on a cloudless day in July 2007 and again in July 2008 using a LI-250 light meter (LI-COR, Lincoln, Nebraska, USA).

Composition of understory vegetation and the percent coverage in the plots where understory vegetation was maintained were visually assessed throughout the study. We randomly selected 8 1 m×1 m subplots in each plantation and measured standing litter biomass at the ground level just before the experiment. All the standing litter samples were taken to the laboratory and oven dried at 65°C to constant mass, and then weighed.

### Data collection and analysis

At the end of the experiment (20 months after transplanting), 10 plants (roots and shoots) per plot were randomly collected if more than 10 seedlings survived; otherwise, all surviving seedlings were collected. Seedlings were individually excavated with a shovel, and separated into leaves, stems, and roots. The harvested plant parts were washed, oven-dried to constant mass at 65°C, and weighed. All the samples were further milled to fine powder and analyzed for total [N] and [P]. After digesting the samples, N and P were determined by colorimetric analysis with an auto-analyzer [24]. The relative growth rate (RGR, cm cm^−1^ day^−1^) of seedling height for each species was calculated as RGR  =  (ln H_2_−ln H_1_)/(t_2_−t_1_), where H_1_ and H_2_ represent the seedling height at the start of the experiment (t_1_) and at the end of the experiment (t_2_), respectively.

The effects of transplanted species, plant tissues and the treatment of understory vegetation and litter on [N], [P] and N∶P ratio were assessed using the split-plot design ANOVAs for each plantation. We also analyzed the effects of transplanted species and treatment of understory vegetation and litter on RGR. Differences in environmental conditions between the two plantations were analyzed using t-test. Variables were log_10_ or arcsine square-root transformed when they did not satisfy normality assumptions. Least Significance Difference (LSD) were used for multiple comparison when ANOVAs were significant at α = 0.05. The relationship between leaf [N], [P], N∶P ratio and RGR of seedlings was determined by Pearson correlation analysis. All analyses were performed using SPSS 13.0 for Windows (SPSS software Inc., USA).

## Results

### Environmental conditions

The environmental variables measured in the two plantations were shown in Table 1. At the EP and the NP, the main understory species were the herbaceous *Ottochloa nodosa* and *Melastoma dodecandrun* with a combined coverage of 29.3%, and *O. nodosa* with a coverage of 40%, respectively. Light penetration to the understory was relatively low and no significant difference was found between the two plantations. The standing litter biomass in the plots at the NP was much higher than that at the EP (*p*<0.05). There was no significant differences in soil organic matter, soil hydrolyzed nitrogen and soil available phosphorus between the two plantations (Table 1).

**Table 1:** The dominant understory species and its coverage, standing litter biomass, soil properties, and light penetration at the two plantations.

Plantation	Dominant understory species	Coverage (%)	Standing litter biomass (g/m^2^)	Light penetration (%)	Soil organic matter (g/kg)	Soil hydrolyzed nitrogen (mg/kg)	Soil available phosphorus (mg/kg)
EP	*Melastoma dodecandrun* + *Ottochloa nodosa*	29.3±5.2a	361±38b	12.4±2.1a	1.38±0.01a	105.8±8.1a	1.97±1.04a
P	*Ottochloa nodosa*	40.0±15.2a	877±107a	9.4±2.2a	1.55±0.10a	101.3±5.9a	1.82±0.40a

Values are means ± standard errors and are based on data collected just before the experiment began and from plots where vegetation and litter were not removed.

Means in a column followed by different letters are significantly different (*p*<0.05) according to t-test. EP, *Eucalyptus* plantation; NP, Native species plantation.

### Plant [N], [P] and N∶P ratio

**Figure 1:**
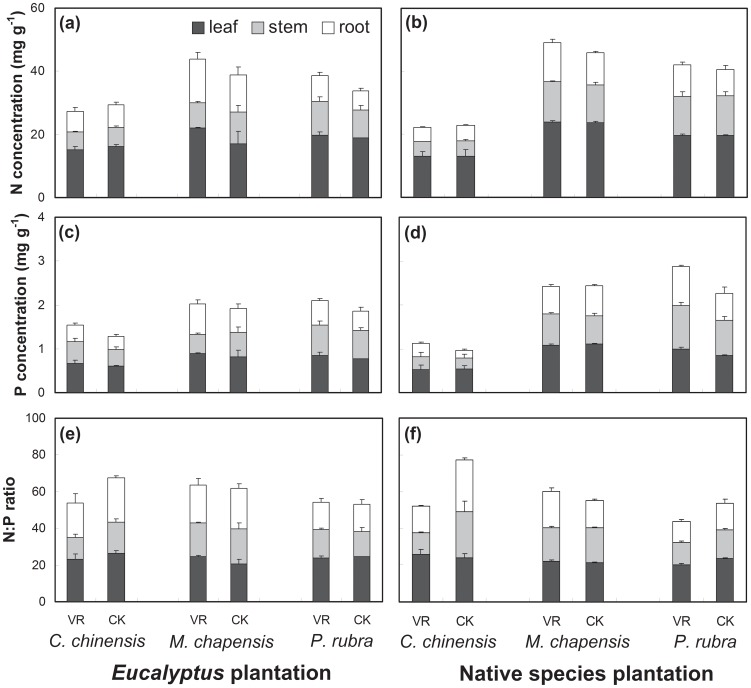
Concentration of nitrogen ([N]), phosphorus ([P]), and N:P ratio in the leaves, stems and roots of *C. chinensis*, *M. chapensis* and *P. rubra* seedlings treated with removal (VR) or retention (CK) of understory vegetation and litter at the two plantations.

At both the EP and the NP, no significant differences in leaf, stem and root [N] and [P] were observed between the VR and the CK treatments for all species, except that root [P] of *C. chinensis* seedlings at the NP (Figure 1d). Generally, at both plantations, [N] and [P] in leaves were much higher than those in roots for all the three species, regardless of understory vegetation and litter treatments.

Plant [N] and [P] were significantly affected by species and plant tissue at both plantations (Table 2). [P] at the NP was also affected by treatment and the interaction between species and plant tissue. At the EP, [N] in the leaves, stems and roots, and [P] in the roots of *M. chapensis* seedlings were higher than that in *C. chinensis* seedlings under the VR treatment (Figure 1 a & c ; *p*<0.05). Under both treatments (VR and CK), [N] and [P] in the leaves, stems and roots of the *C. chinensis* seedlings were much lower than the other two species at the NP (Figure 1 b & d; *p*<0.05).

**Table 2:** ANOVAs for the effects of treatment (removing or retaining understory vegetation and litter), species and plant tissue on the concentration of nitrogen ([N]), phosphorus ([P]), and N∶P ratio at the two plantations.

Source of Variation	*df*	[N]		[P]		N∶P ratio	
		*F*	*p*	*F*	*p*	*F*	*p*
*Eucalyptus* plantation							
Treatment (T)	1	2.084	0.156	3.400	0.072	0.713	0.403
Species (S)	2	10.273	**0.000**	11.380	**0.000**	3.865	**0.029**
Plant tissue (PT)	2	67.433	**0.000**	23.406	**0.000**	15.560	**0.000**
T×S	2	1.469	0.242	0.247	0.782	2.774	0.074
T×PT	2	0.911	0.410	0.514	0.602	0.678	0.513
S×PT	4	2.363	0.068	2.339	0.071	2.060	0.103
T×S×PT	4	0.626	0.647	0.451	0.771	0.151	0.962
Native species plantation							
Treatment (T)	1	1.819	0.184	12.872	**0.001**	9.764	**0.003**
Species (S)	2	90.766	**0.000**	103.731	**0.000**	7.741	**0.001**
Plant tissue (PT)	2	146.099	**0.000**	29.763	**0.000**	12.876	**0.000**
T×S	2	1.547	0.223	2.804	0.070	8.192	**0.001**
T×PT	2	0.456	0.636	0.252	0.778	2.229	0.118
S×PT	4	2.100	0.095	4.030	**0.007**	1.062	0.385
T×S×PT	4	0.192	0.941	1.064	0.384	3.097	**0.024**

*F*-ratios and significance values for treatment, species, plant tissue and their interactions are given (significant results in *bold*).

The N∶P ratio was significantly affected by species and plant tissue at two plantations (Table 2). N∶P ratio at the NP was also affected by the treatment, the interaction between species and treatment, and the interaction among treatment, species and plant tissue. The values of N∶P ratio ranged between 11.2 and 27.9 for all plant tissues and species at the two plantations. The VR treatment significantly reduced the leaf and stem N∶P ratio in *P. rubra* seedlings and the root N∶P ratio in *C. chinensis* seedlings at NP (Figure 1f; P<0.05). At the EP and the NP, stem N∶P ratio in *C. chinensis* seedling was much lower than that in *M. chapensis* under the VR treatment (Figure 1 e & f; *p*<0.05), while the root N∶P of *C. chinensis* seedling was much lower than that of *P. rubra* under the CK treatment (Figure 1 e & f; *p*<0.05).

### Relative growth rate (RGR) of seedling height, and its relationship with N, P concentration and N∶P ratio

**Figure 2:**
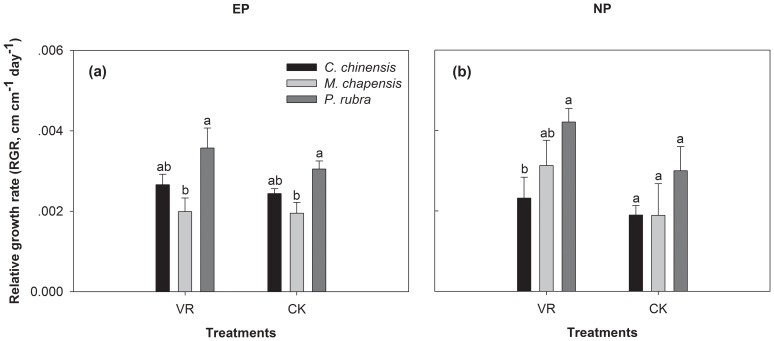
Relative growth rate (RGR) of height for transplanted *C. chinensis*, *M. chapensis*, and *P. rubra* seedlings affected by removing (VR) or retaining (CK) understory vegetation and litter at the two plantations. EP: *Eucalyptus* plantation; NP: native species plantation.

For both plantations, relative growth rate (RGR) of seedling height was significantly affected by the introduced species. At the EP, RGR of *M. chapensis* seedling was much lower than that of *P. rubra* seedlings, regardless of whether understory vegetation and litter were present or absent (Figure 2a). When removing understory vegetation and litter at the NP, RGR of *P. rubra* seedlings was higher than that of *C. chinensis* seedlings (Figure 2b). No significant difference in RGR was detected between the treatments of understory vegetation and litter for all the three species (Figure 2)

**Figure 3:**
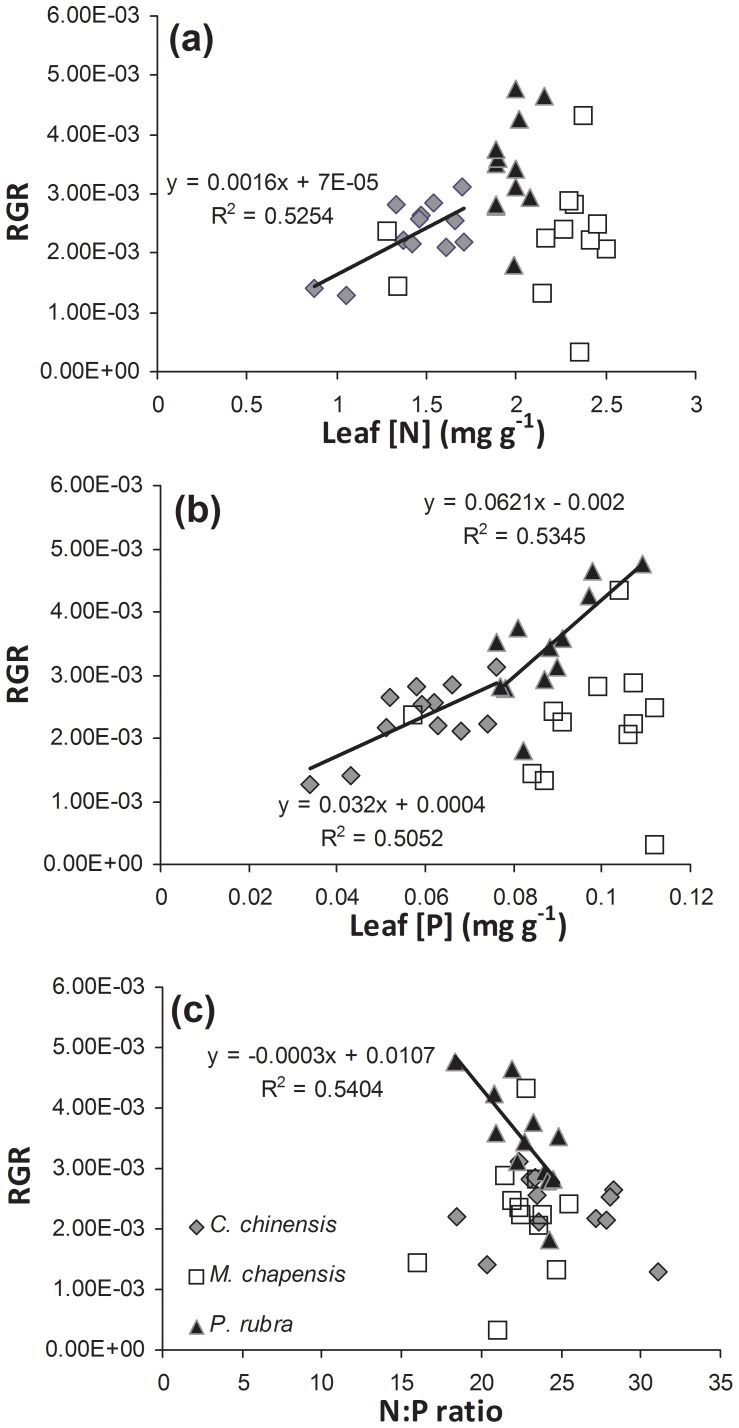
Relationship between RGR of height and leaf [N], [P] and N∶P ratio for the transplanted *C. chinensis*, *M. chapensis*, and *P. rubra* seedlings.

For *C. chinensis*, RGR was positively correlated with leaf [N] and [P] (Figure 3a&b r = 0.725, *p* = 0.008, and r = 0.711, *p* = 0.01, respectively). For *P. rubra* seedlings, RGR was also positively correlated with leaf [P] (Figure 3b; r = 0.732, *p* = 0.007), but negatively correlated with leaf N∶P ratio (Figure 3c; r = −0.736, *p* = 0.006). No significant relationship was found between RGR and leaf [N], [P] or N∶P ratio for *M. chapensis* seedlings.

## Discussion

### Effect of understory vegetation and litter on seedling leaf [N] and [P], and P∶N ratio

Understory vegetation was indicated as a competitor of resources in the field, and controlling understory vegetation could increase nutrient concentrations of planted seedlings [14]. Contrary to previous reports, our results showed that removing understory vegetation and associated litter had no effect on seedling [N] and [P]. In our study, the understory vegetation is mainly composed of herbaceous species (*M. dodecandrun* and *O. nodosa*) with low coverage (Table 1). Therefore, the competition from the understory vegetation may not reach the level necessary to strongly influence the nutrient concentrations in the seedlings. Meanwhile, the existence of litter on the floor may alleviate the influence of understory vegetation on the introduced seedlings, due to increasing soil moisture and releasing nutrients to the soil through litter decomposition [25], [26]. In the current study, understory vegetation and litter were considered as a combined factor and therefore the effects of understory vegetation could not be separated from the effect of litter. The treatment of understory vegetation and litter removal may also reduce the plant litter nutrient release to the soil.

Our results showed that leaf [N] and [P], and P∶N ratio generally positively correlated with RGR in height of transplanted seedlings, consistent to previous studies [7], [27]. For terrestrial vascular plant species, RGR was positively correlated with leaf [N] and [P], and negatively with leaf N∶P ratio. But such correlation may not necessarily be applicable to plants grown at high nutrient supply [8]. Because of the low soil P availability in the experimental subtropical plantations, the relationship between RGR and leaf [N], [P] and N∶P ratios observed in our study is reasonable.

### Variations in plant tissue [N] and [P] among species

The ability of plants to accumulate mineral nutrients is probably genetically controlled and thus can be ascribed to the influence of species [15]. In our study, [N] and [P] in all the plant tissues of *C. chinensis* seedlings were relatively lower compared to these in the other two transplanted species, indicating an inferior nutrient uptake capability. Root length is a major factor governing which plants are more successful in competing for soil nutrients [28]. In this respect, we were surprised that *C. chinensis*, which is a fast-growing species with long tap roots, had lower nutrient concentration in the tissues. The reason was probably related to its shade tolerance. There is evidence that shade tolerance of species is correlated with plant nutrient status and foliar nutrition is positively related to light conditions for shade-intolerant species due to reduced nutrient uptake under shady environment [29], [30]. In our experimental plantations especially the native species plantation, the light penetration to the understory is low (Table 1), which may partially explain the lower plant nutrition of the shade-intolerant *C. chinensis* compared to the intermediate shade-tolerant *M. chapensis* and the shade-tolerant *P. rubra*.

### Implication for regeneration management

Soil nutrient status directly influences plant nutrient concentration. Understanding the plant nutrient-soil relationship can provide a useful guide for suitable fertilizer requirements to improve initial seedling growth [27], [31], [32]. P limitation in our study is reflected by the lower [P] (varying between 0.18–1.11 mg g^−1^), as [P] under optimal growth conditions vary between 2–5 mg g^−1^ [11]. The N∶P ratios, ranged between 11.2–27.9 in the transplanted seedlings, also indicate that plant growth might be limited by soil N and P, particularly P [8]. Such observation was consistent with previous findings that the soil in southern China had low P content [33].

For better regeneration management in the established plantations, it appears that the addition of phosphorus fertilizer is essential to enhance seedling growth and performance of the transplanted indigenous tree species. Meanwhile, better site preparation such as controlling understory standing vegetation may be important as fertilization of P has a greater positive effect on plant [P] when competing vegetation is removed [34], even though removing understory vegetation had no significant effects on [N] and [P] of transplanted seedling in the current study.

## Conclusions

By transplanting different indigenous tree species into subtropical plantations, we demonstrated that species selection could be an important factor determining leaf nutrient concentrations and potential survive ability in the subtropical *Eucalyptus* plantation and native species plantation. Compared to the *C. chinensis*, *M. chapensis* and *P. rubra* seem superior in macro-nutrient accumulation. It is surprising that no significant effects of understory vegetation and litter removal were found on plant tissue [N], [P] and N∶P ratio, and RGR of seedlings. This may be due to the lower competition ability of nutrient uptake by understory vegetation and more nutrients released from litter to the soils. The relationship between RGR of seedling and leaf [N], [P], and N∶P ratio varied among introduced species. RGR was positively correlated with leaf [N] and [P], but negatively correlated with leaf N∶P ratios, indicating that plant growth might be limited by soil nutrients, particularly P. A better understanding of N or P limitation for seedling growth in the experimental plantations will require a further N- and P- addition experiments in the field.
